# Contrast-induced nephropathy in patients with diabetes mellitus between iso- and low-osmolar contrast media: A meta-analysis of full-text prospective, randomized controlled trials

**DOI:** 10.1371/journal.pone.0194330

**Published:** 2018-03-20

**Authors:** Xiao-fang Han, Xin-xiu Zhang, Ke-mei Liu, Hua Tan, Qiu Zhang

**Affiliations:** 1 Department of Endocrinology, The First Affiliated Hospital of Anhui Medical University, Hefei, China; 2 Department of Endocrinology, The Second People's Hospital of Hefei, Anhui, China; 3 Center for Bioinformatics & Systems Biology, Department of Radiology, Wake Forest School of Medicine, Winston Salem, NC, United States of America; Istituto Di Ricerche Farmacologiche Mario Negri, ITALY

## Abstract

**Purpose:**

This study was conducted to compare iso-osmolar contrast medium, iodixanol, with low-osmolar contrast media (LOCM) for assessing contrast-induced nephropathy (CIN) incidence, exclusively in the diabetic population.

**Method:**

A systematic search was conducted for full-text, prospective, randomized controlled trials (RCTs). The primary outcome was incidence of CIN. Medline, Cochrane Central Register of Controlled Trials, and other sources were searched until May 31, 2017.

**Results:**

Twelve RCTs finally met the search criteria. Iodixanol did not significantly reduce the risk of CIN (risk ratio [RR]: 0.72, 95% confidence interval (CI): [0.49, 1.04], p = 0.08). However, there was significantly reduced risk of CIN when iodixanol was compared to a LOCM agent iohexol (RR: 0.32, 95% CI [0.12, 0.89]). There were no differences between iodixanol and the other non-iohexol LOCM (RR: 0.92, 95% CI [0.68, 1.25]).

**Conclusion:**

In diabetic populations, iodixanol is not associated with a significant reduction of CIN risk. Iodixanol is associated with a reduced risk of CIN compared with iohexol, whereas no significant difference between iodixanol and other LOCM could be found.

## Introduction

Contrast-induced nephropathy (CIN), is an acute impairment in renal function, and typically occurs within 3 days following the exposure of a contrast medium (CM) [1–3]. In the United States, CIN is one of the leading causes of acute kidney injury, accounting for 11–14.5% [4,5], and is associated with increased cost, hospital stay, and long-term morbidity and mortality [6–8].

Patients at highest risk for CIN include these with pre-existing renal injury, particularly when it is secondary to diabetic nephropathy (DN). It has been recognized that adults with diabetes have a higher risk of developing DN than those without this disease [9,10]. A milestone meta-analysis [11] mentioned the independent predictors of CIN included chronic kidney disease (CKD), CKD with DM, and use of LOCM. However, CKD with DM group had a much higher increase of maximum serum creatinine (SCr), than CKD without DM after contrast exposure. Also, paramount to say that this meta [11] extracted more data from internal database of GE Healthcare, the manufacturer of iso-osmolar contrast media iodixanol. Besides, some evidences investigated the diabetic patients may have some degree of reduced renal function despite having normal SCr levels [12–14]. And, a pilot small RCT [15] proved that in diabetic patients, iodixanol was associated with a lower incidence of CIN than low-osmolar ioversol. So far, it has no systemic reviews or meta-analyses to compare iodixanol with LOCM, exclusively in the diabetic population *per se* with or without renal insufficiency.

This meta-analysis will expand prior analyses by incorporating all fully-published, prospective RCTs. More specifically, our study will focus on the diabetic population with or without CKD to provide a comparison for nephrotoxicity between the iso-osmolar agent iodixanol and the LOCM.

## Methods

### Data sources and search strategy

A computerized search to identify eligible RCTs was conducted using the databases of Medline (via PubMed), Cochrane Central Register of Controlled Trials (CENTRAL, 1999–2017 John Wiley & Sons, Inc.), the internal database of GE Healthcare, the manuscript of iodixanol as well as references of retrieved articles and prior meta-analyses, using the following keywords: “Contrast-induced nephropathy,” “contrast-induced acute kidney injury,” “contrast medium,” “iodixanol,” “visipaque,” “iso-osmolar,” “low-osmolar,” “nephropathy,” “nephrotoxicity” and “renal dysfunction”.

### Study selection

RCTs were included of comparing CIN events in diabetic patients who were given iso-osmolar iodixanol versus LOCM. Studies were eligible for inclusion if they prospectively randomized patients to either iodixanol or at least one kind of LOCM, and if data on renal function or CIN was routinely ascertained. The primary outcome was the incidence of CIN, defined as an absolute increase at least 0.5 mg/dL (44.2 μmol/L) or relative increase at least 25% from baseline value of SCr, based on current recommendations by the European Society of Urogenital Radiology (ESUR) in the year of 1999 [1] and 2011[2] as well as the Canadian Association of Radiologists [3]. Only fully-published, prospective, RCTs were included for quantitative assessment. Studies that were retrospective, non-randomized or those in which patients were not randomized to either iodixanol or LOCM were excluded strictly. Exclusion also applied to those without available data regarding the patients with diabetes mellitus. No restrictions were placed on languages or sample sizes.

### Quality assessment

We used the Cochrane Collaboration’s recommended tool for assessing the risk of bias in included studies [16]. To determine an overall quality of included studies, we assessed trial’s quality by evaluating every element of study design (i.e., blinding description, randomization process, inclusion and exclusion criteria, concealed allocation, intention-to-treat analysis, and assessment of withdrawals and dropouts), together with Jadad scoring system. Risk for bias was assessed in duplicate, with disagreements resolved by consensus. Potential publication bias was further assessed by Begg’s funnel plot [17].

### Data extraction

Data were independently extracted by two reviewers (HX, ZX), and disagreements were resolved by consensus with the third reviewer (LK). The primary outcome, incidence of CIN, was extracted. Baseline demographic, clinical, and procedural characteristics were recorded, including patients’ characteristics (i.e. age), sample sizes, type of LOCM (i.e. iohexol, S1 Table), iodine concentration (i.e. iohexol 300 mg I/mL) and time frame of CIN after exposure of contrast was also extracted.

### Data analysis

Analyses were mainly performed with Review Manager Software (RevMan Analyses Version 5.3.Copenhagen; The Nordic Cochrane Center, The Cochrane Collaboration, 2014). For dichotomous variables, the risk ratio (RR) was measured along with a 95% confidence interval (CI). A 95% CI not including 1 or p<0.05 was considered to be statistically significant. The inter-study statistical heterogeneity was examined by both Chi-squared tests and I^2^ statistics. A random-effects model was performed to attain a more relative conservative result. To explore sources of heterogeneity, each study was removed one by one to detect its contribution. If removal of the studies using this process did not create a statistical significant change, the results would be considered robust. Potential publication bias and skewness were assessed graphically using funnel plots in RevMan, if the included studies were greater than or equal to 10.

Subgroup analyses were also conducted based on the formulations of LOCM, which were also divided as iohexol group and non-iohexol group. All CIN events were incorporated, in spite of variant definitions of CIN. Thus, 4 studies [18–21] supplied available data of both two CIN definitions. In this study, we named, i.e. *Chen (1) 2012* that meant Chen 2012 [19] with the data of relative CIN criterion, while the *Chen (2) 2012* meant the absolute criterion.

## Results

### Study selection

Medline (via PubMed), CENTRAL (search criterion in S2 Table) and other sources were searched from inception to May 31, 2017.

**Fig 1:**
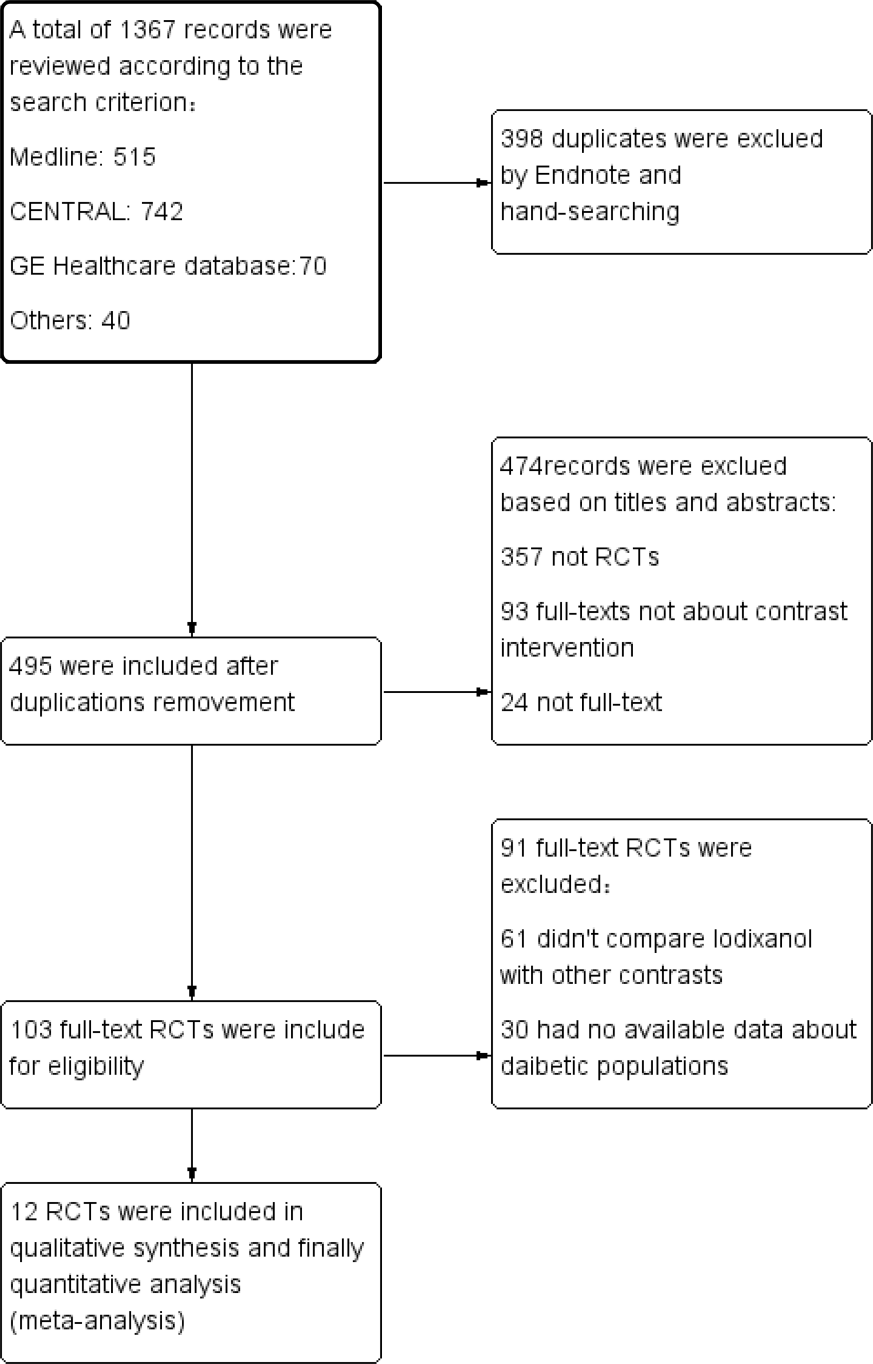
Flow diagram of selection strategy. Flow diagram depicting the selection strategy for trials used in this meta-analysis. 12 RCTs finally met our criteria. RCT = randomized clinical trials.

A total of 1367 potentially relevant studies were identified. Of these, 398 were duplicates and 474 had no association or available data about CIN based on review of title and abstract. Of the 103 remaining studies for full-text assessment, 61 didn’t compare iodixanol with LOCM and the other 30 were excluded as they had no available data individually about diabetic populations. Finally, 12 prospective full-text trials [15,18–28] were included in the process of qualitative analysis (Fig 1). Demographic and baseline characteristics of the 12 trials were summarized (Table 1). Elements of study quality were listed as blinding description, randomization process, inclusion and exclusion criteria, concealed allocation, intention-to-treat analysis and assessment of withdrawals and dropouts. And the total quality was assessed by Jadad scores (S3 Table). Among these studies, all were prospective, double-blind RCTs with a score greater than 3 scores, other than Hernandez 2009[15], a single-center, open-label but prospective, randomized study. The study was also consistent with the protocol of PRISMA [29] (**P**referred **R**eporting **I**tems for **S**ystematic reviews and **M**eta-**A**nalyses statement, S4 Table).

**Table 1:** Characteristics of trials.

First Author	publish Year	Acronym	Patients’ charecteristic	Primary outcome*	LOCM^†^	Patients, n			Mean age‡		Male, (%)		DM, (%)	
						Total	Iodixanol	LOCM	Iodixanol	LOCM	Iodixanol	LOCM	Iodixanol	LOCM
Aspelin	2003	NEPHRIC	DM and CKD	peak increase of SCr between day 0 and 3	iohexol, 350	129	64	65	71.1±6.0	70.6±8.6	64	53.8	100	100
Jo	2006	RECOVER	CKD	SCr≥0.5 or ≥25% on day 1–2	ioxaglate, NR	275	140	135	66.1±8.6	68.7±7.5	56.4	55.6	34.3	36.3
Feldkamp	2006	NR	normal renal function	SCr≥25% or CrCl <20%	iopromide, 300	221	105	116	60.6±10.0	62.1 ± 9.2	75.2	75.9	21.9	20.7
Solomon	2007	CARE	CKD	SCr≥0.5 in 45–120 hrs	iopamidol, 370	414	210	204	70.5±9.9	72.4±9.0	60.5	67.6	43.8	38.2
Rudnick	2008	VALOR	CKD	SCr>0.5 within 3d	ioversol, 320	299	156	143	71.1 ± 9.9	72.6 ± 10.2	68	74	52	52
Hardiek	2008	NR	DM and CKD	SCr≥25%	iopamidol, 370	102	54	48	65±10	66±10	48	67	100	100
Kuhn	2008	PREDICT	DM and CKD	SCr≥25% on day 2–3	iopamidol, 370	248	123	125	68.3 ± 9.19	69.5 ±10.05	43.2	50.4	100	100
Hernández	2009	NR	DM	SCr≥0.5 or 25% on day 3	ioversol, 350	250	118	132	69.1±9.0	70.1±7.9	61.9	64.4	100	100
Laskey	2009	NR	CKD, DM	SCr≥0.5 within 3d	iopamidol, 370	526	263	263	69.5±8.7	69.8±8.9	67	66	100	100
Chuang	2009	NR	CKD with or with DM	SCr≥25% with 7 days	Iohexol, NR	50	25	25	62.9 ± 13.7	53.0 ± 12.2	72	64	40	36
Shin	2011	NR	CKD	SCr≥0.5 or 25% after the procedure	iopromide, 300	420	215	205	71.1±8.7	71.9±8.2	57	51	44	49
Chen	2012	DIRECT	CKD	SCr≥50% on day 3	iopromide, 370	562	284	278	70.0±9.25	69.0±10.5	66.9	67.6	32.7	27.7

*: mg/dL is used to calculate SCr; mL/min to CrCl

†: mg I/ml to LOCM

‡: Values reported as mean±SD

LOCM = low-osmolar contrast media; DM = diabetes mellitus; CKD = chronic kidney disease; SCr = serum creatinine; CrCl = creatinine clearance; NR = nor reported.

### Data extraction

In the included studies, the incidence of CIN was reported. Among all studies, only five [15,18,20,23,28] recruited the diabetic populations. As for the remaining 7 studies [19,21,22,24–27], the available data of CIN in the diabetic patients were listed as a subgroup and disclosed in the main text. All data were extracted from the published articles directly.

### CIN definition

The primary outcome of our study was the incidence of CIN, defined as a relative increase in the SCr of at least 25% or an absolute increase of at least 0.5 mg/dl from the baseline value after contrast medium exposure. Among all studies, 4 trials [20,26–28] defined CIN as SCr ≥25% as the primary clinical endpoint, 3 defines as SCr ≥0.5 mg/dl [21,23,24], and another 3 either [15,22,25]. Besides, 4 studies [18–21] also supplied other available data of CIN other than the primary outcome defined individually in each study.

### Relative criterion of CIN

Available data from 7 studies [18–21,26–28] disclosed the incidence of CIN after contrast media exposure, which was defined as a relative increase in the SCr of at least 25% from the baseline.

**Fig 2:**
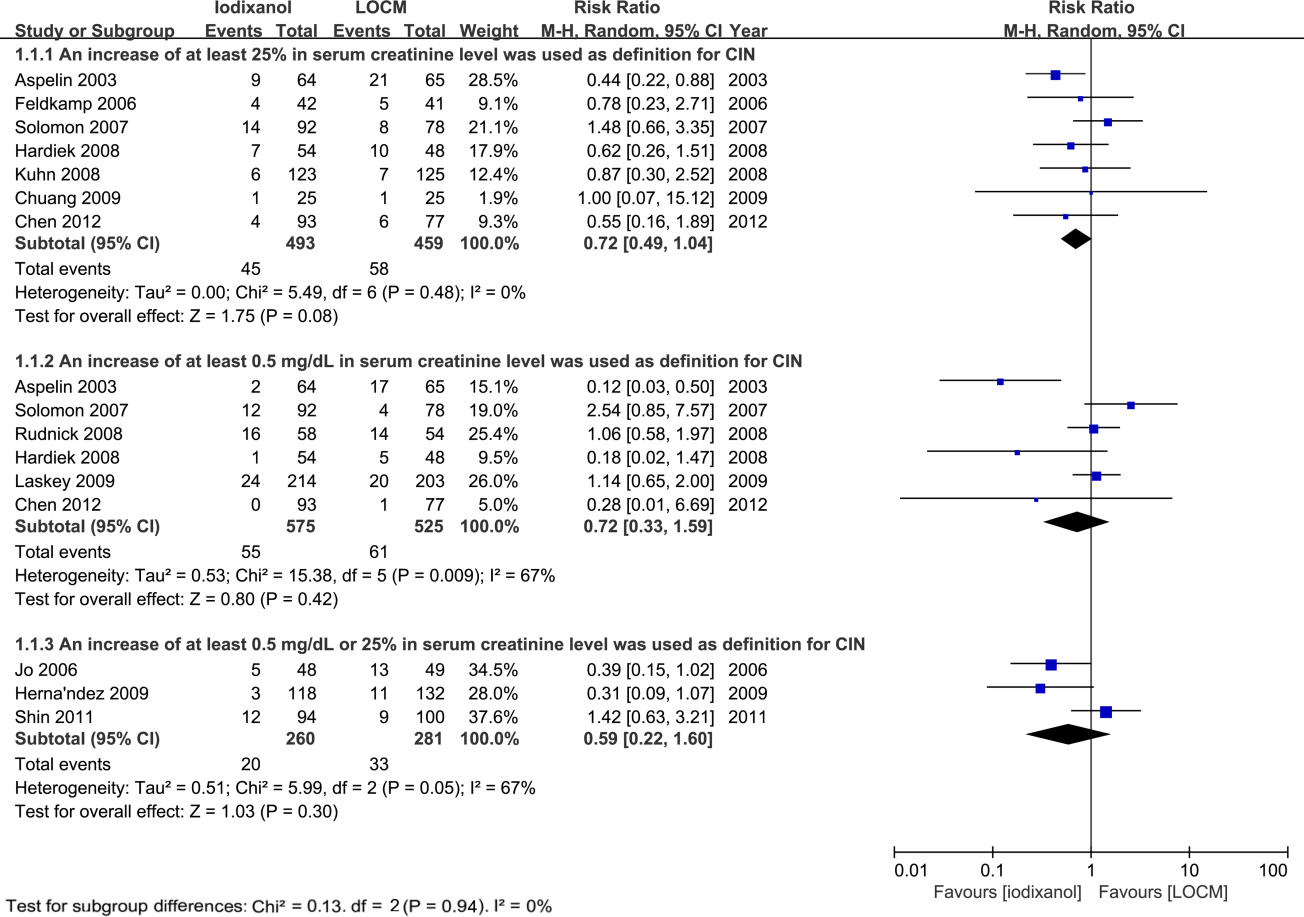
RR of CIN for comparison of iodixanol with LOCM in diabetic patients. Forest plot of risk ratios (RR) for the incidence of contrast-induced nephropathy (CIN) for comparison of iodixanol with low-osmolar contrast media (LOCM). CIN was defined as an increase of more than 25%, or 0.5 mg/dL (44.2 μmol/L), or either, in serum creatinine level (SCr), respectively. LOCM low-osmolar contrast medium/media; CI confidence interval; M-H Mantel-Haenszel method.

In total, CIN occurred among 45 out of 493 who received iodixanol, and 58 of 459 patients who received LOCM (n = 952, RR: 0.72, 95% confidence interval [CI]: 0.49–1.04, p = 0.08, I^2^ = 0%, Fig 2), indicating that there was slight reduction of incident CIN associated with iodixanol in comparison to LOCM, but insignificantly.

However, the nonsignificant result wasn’t robust after removing the study of Solomon 2007[21] (RR: 0.59, 95% CI: 0.59–0.90, p = 0.01, I^2^ = 0%).

### Absolute criterion of CIN

Separately, available data from 6 studies [18–21,23,24] uncovered the incident CIN defined as an absolute increase in the SCr of at least 0.5 mg/dl after given contrast media.

Totally, the events of CIN occurred among 55 out of 575 who received iodixanol, and 61 of 525 patients who received LOCM (n = 1100, RR: 0.72, 95% CI: 0.33–1.59, p = 0.42, Fig 2), indicating that there was no significant difference of incident CIN associated with iodixanol in comparison to LOCM, with a higher heterogeneity (p = 0.009, I^2^ = 67%).

Furthermore, the results were robust after removing each study one by one to detect its contribution without a statistically significant change.

### Relative or absolute definition of CIN

Only 3 studies [15,22,25] defined the CIN as SCr≥25% or ≥0.5 mg/dl, without other available data separately. In total, CIN occurred among 20 out of 260 who received iodixanol, and 33 of 281 patients who received LOCM (RR: 0.59, 95% CI: 0.22–1.60, p = 0.30, Fig 2), indicating that there was no significant reduction of CIN rate associated with iodixanol in comparison to LOCM.

### Iodixanol versus LOCM

**Fig 3:**
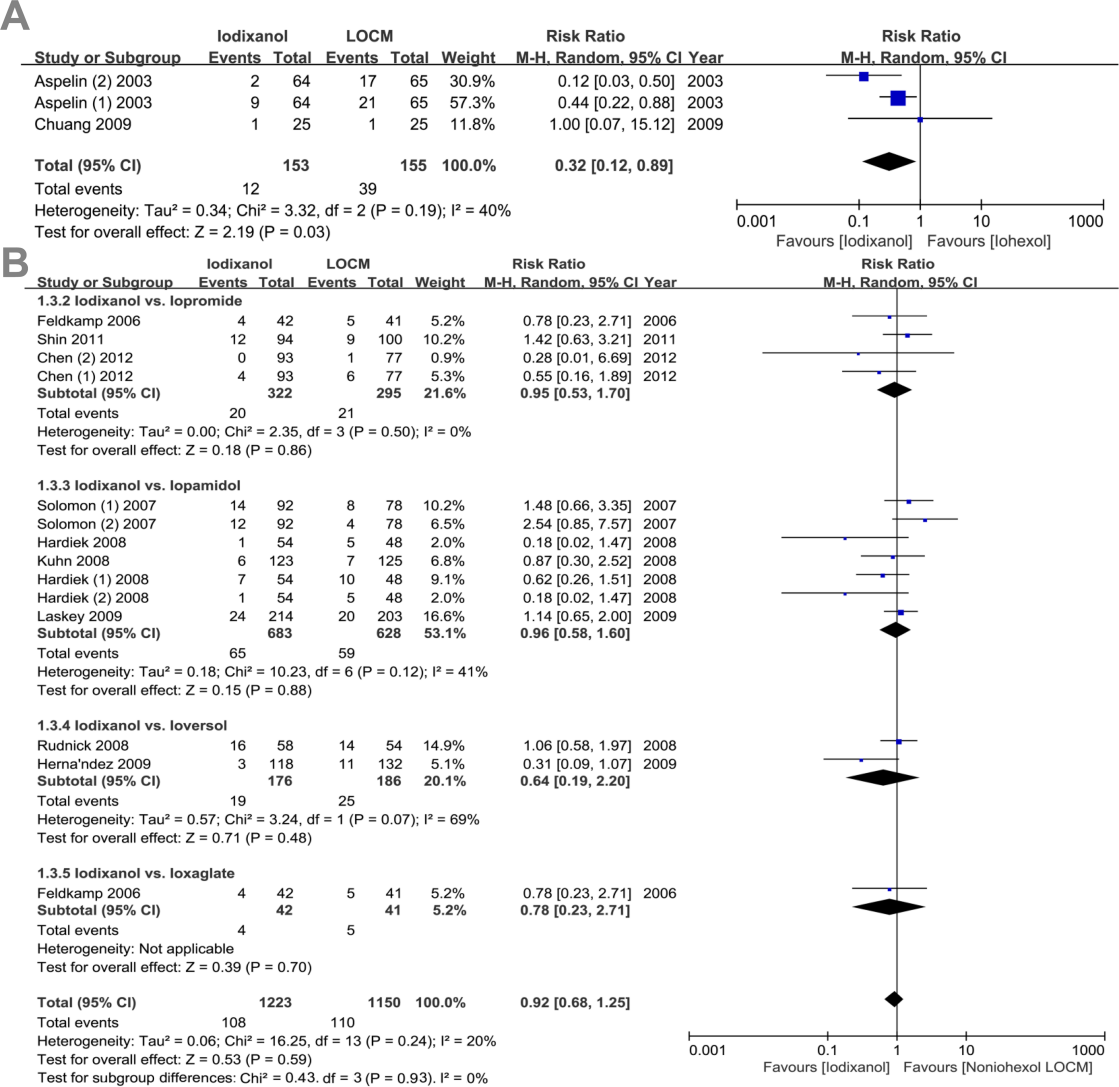
RR of CIN for comparison of iodixanol with iohexol or other non-iohexol LOCM. Funnel plot of studies for contrast-induced nephropathy (CIN) for comparison of iodixanol with iohexol or other non-iohexol LOCM in diabetic patients.

Subgroup analyses were conducted based on the formulations of contrast media (Fig 3). Patients receiving nonionic, monomeric LOCM were given iohexol (3 trials), iopamidol (7 trials), iopromide (4 trials), ioversol (2 trial), as well as ionic dimer, ioxaglate (1 trial).

There was a significant reduction of CIN when iodixanol was compared to iohexol (n = 308, RR: 0.32, 95% CI: 0.12 to 0.89, p = 0.03; I^2^ = 40%), iopromide (n = 617, RR: 0.95, 95% CI: 0.53 to 1.70, p = 0.86) and iopamidol (n = 1,311, RR: 0.96, 95% CI: 0.58 to 1.60, p = 0.88). However, there was no significant difference between iodixanol and iopromide (n = 617, RR: 0.95, 95% CI: 0.53 to 1.70, p = 0.86) and iopamidol (n = 1,311, RR: 0.96, 95% CI: 0.58 to 1.60, p = 0.88). Furthermore, when iodixanol was compared to non-iohexol LOCMs, there was also no significant (n = 2,375, RR: 0.92, 95% CI: 0.68 to 1.25, p = 0.93).

## Discussion

### Major findings

From including 12 prospective full-text RCTs to compare iso-osmolar CM, iodixanol, with LOCM for assessing the incidence of CIN exclusively in the patients with diabetes mellitus, numerous findings were found as follows: 1) iodixanol isn’t superior to LOCM to reducing the risk of CIN; 2) If the CIN is defined as a relative increase in SCr of at least 25% from baseline, iodixanol showed slightly reduced CIN incidence non-significantly. However, the result seems not very robust; 3) iodixanol is superior to iohexol to reduce the risk of CIN; 5) No significant difference between iodixanol and other non-iohexol LOCMs could be found

### Definitions of CIN

CIN was first defined in 1999 by ESUR [1] as an absolute increase in SCr of at least 0.5 mg/dl or a relative increase of >25% of baseline value. Although this definition was followed by some standard guidelines [2,3], a series of derivative definitions were still widely used in recent years, i.e. both absolute and relative increase, absolute increase solely, relative increase solely. Moreover, the upper limitations of SCr increase were not mandatory, which also deteriorated the consistency of criterion for CIN. For example, Chen 2012 [19] defined CIN as SCr increase ≥50%. Variant definitions brought inconsistency, even in the meta-analyses of RCTs. McCullough [11] firstly defined the CIN as SCr increase ≥0.5 mg/dl and found the iodixanol was associated with a reduced of CIN. While, From et al. [30] found that iodixanol was not associated with less CIN.

For avoiding the inconsistency of variant definitions, uniform criteria were conducted in this study. Also, the more widely-used definitions of SCr increase ≥25% or 0.5 mg/dl were adopted.

### The incidence of CIN in diabetic populations

There is general agreement that CKD is the most significant risk factor for CIN and every multivariate analysis has shown that CKD is an independent risk factor for CIN [4,31–35]. A recently published meta-analysis reported a very closely related study, albeit examining chronic kidney disease patients rather than diabetic patients. Randomized trials comparing IOCM to LOCM in CKD stage 3 and above patients undergoing CA, and reporting incidence of CIN (defined by a rise in creatinine of 25% from baseline) were included in the analysis. In patients with CKD stage 3 and above undergoing coronary angiography, use of IOCM showed overall non-significant difference in incidence of CIN compared to LOCM. The difference was further attenuated when IOCM was compared with non-ionic LOCM [33]. Additionally, whether DM could be another independent risk factor for CIN is controversial. There is no conclusive evidence that diabetic patients weren’t at an increased risk of CIN if their renal function is normal [2]. The finding of this study indicated that iodixanol was not associated with a reduced risk of CIN, was based on the uniform definitions of CIN respectively, which indicated that DM *per se* wasn’t independent predictors of CIN.

### Iodixanol versus LOCM

Three prior meta-analyses, Reed 2009[36], Heinrich 2009[37] and From 2010[38] also found that iodixanol was associated with a reduction in CIN compared to iohexol, although significant differences between iodixanol and non-iohexol nonionic LOCM were inconsistent. Reed [36] reported that iodixanol was superior to ioxaglate. However, Heinrich [37] and From [38] indicated that iodixanol was not associated with a reduction in CIN when compared to non-iohexol.

This study indicated that in patients with diabetes mellitus, iodixanol wasn’t associated with significant renal protection when compared to nonionic LOCM other than iohexol. This finding couldn’t be explained solely by osmolality [18,37,38], which are warranted for further exploration of other possible mechanisms.

### Study strengths and limitations

**Fig 4:**
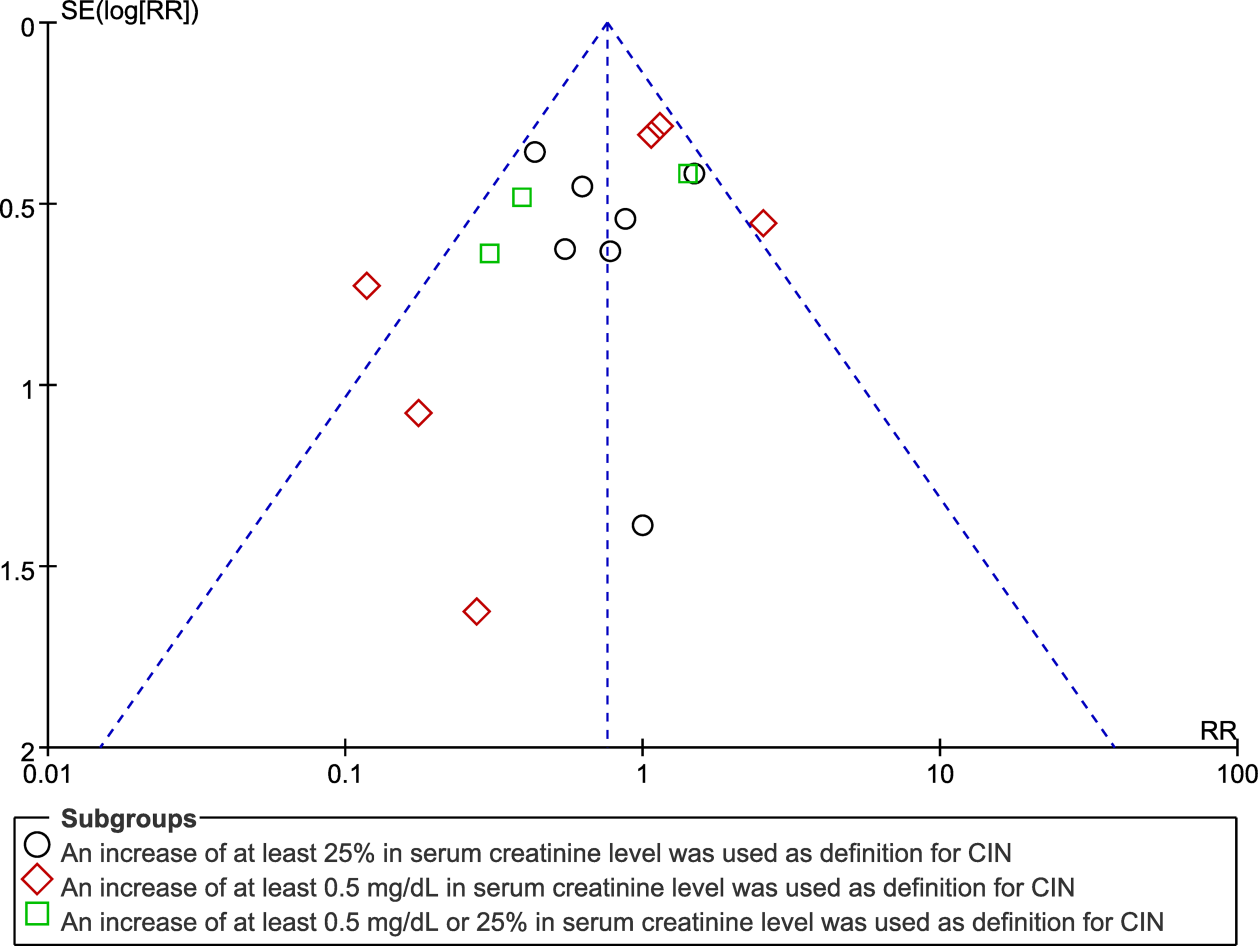
Funnel plot for the association between contrast use and CIN under different definitions of CIN.

One limitation of our study is that the results are based on the comprehensive data of trials with heterogeneous RCTs. The included trials utilized different radiographic procedures, contrast loads, heterogeneous definitions of CIN, or different complications of diabetic patients (CKD or not). We have attempted to account for these differences using random-effects models to obtain more conservative results. A significant strength of our study is the inclusion of mainly relevant RCTs on CIN, divided by each definition. In addition, Begg’s funnel plot analysis results (Fig 4) showed negligible publication bias in our study under all three definitions of CIN, demonstrating that our conclusions are reliable.

## Supporting information

S1 Table Characteristics of different contrast media of our studies. (DOCX)

S2 Table Search criterion of Medline (via PubMed), from inception to May 31, 2017; search criterion of cochrane Central Register of controlled trials, from inception to May 31, 2017. (DOCX)

S3 Table Quality assessment of trials. (DOCX)

S4 Table The study was also consistent with the protocol of PRISMA. (DOC)

## Abbreviations

CIN: Contrast-induced nephropathy
CM: contrast medium/media
CI: confidence interval
CKD: chronic kidney disease
DM: diabetes mellitus
DN: diabetic nephropathy
ESUR: European Society of Urogenital Radiology
LOCM: low-osmolar contrast medium/media
PRISMA: Preferred Reporting Items for Systematic reviews and Meta-analyses statement
RCT: randomized controlled trials
RR: risk ratio
SCr: serum creatinine
