# Supplementary material for: Contrast-induced nephropathy in patients with diabetes mellitus between iso- and low-osmolar contrast media: A meta-analysis of full-text prospective, randomized controlled trials
Source: PLoS One. 2018 Mar 20;13(3):e0194330. doi: 10.1371/journal.pone.0194330 (PMC5860737; doi:10.1371/journal.pone.0194330)
Supplement: S1 Table — (DOCX) [file pone.0194330.s001.docx]

**S1 Table. Characteristics of Different Contrast Media of Our Studies**

| **Generation** | **Class** | **Type of Molecule** | **Example** | **Iodine**  **(mg I/ml)** | **Osmolality**  **(mOsm/kg-H_2_O)** |
| --- | --- | --- | --- | --- | --- |
| Second | Low-osmolar | Ionic dimer | Ioxaglate | 320 | 600 |
| Second | Low-osmolar | nonionic Monomer | Iopamidol | 270,300,370 | 796 |
| Second | Low-osmolar | nonionic Monomer | Iohexol | 300 or 350 | 844 |
| Second | Low-osmolar | nonionic Monomer | Iopromide | 370 | 607 |
| Second | Low-osmolar | nonionic Monomer | Iomeprol | 320,350,400 | 726 |
| Second | Low-osmolar | nonionic Monomer | Ioversol | 320 | 792 |
| Third | Iso-osmolar | nonionic Monomer | Iodixanol | 270 or 320 | 290 |
