# Supplementary material for: Contrast-induced nephropathy in patients with diabetes mellitus between iso- and low-osmolar contrast media: A meta-analysis of full-text prospective, randomized controlled trials
Source: PLoS One. 2018 Mar 20;13(3):e0194330. doi: 10.1371/journal.pone.0194330 (PMC5860737; doi:10.1371/journal.pone.0194330)
Supplement: S2 Table — (DOCX) [file pone.0194330.s002.docx]

**S2 Table. Search criterion of Medline(via PubMed), from inception to May 31, 2017**

| **No.** | **Query Results** | **Results** |
| --- | --- | --- |
| [#1](http://www.ncbi.nlm.nih.gov/pubmed) | Search **(((random*[Title/Abstract]) OR ("Randomized Controlled Trials as Topic"[Mesh] OR "Randomized Controlled Trial" [Publication Type]))) AND (((((((((nephrotoxicity[Title/Abstract]) OR nephrotoxic*[Title/Abstract]) OR nephropath*[Title/Abstract]) OR renal dysfunction[Title/Abstract]) OR renal failure[Title/Abstract])) OR (((((((Contrast-induced nephropathy[Title/Abstract]) OR Contrast induced nephropathy[Title/Abstract]) OR contrast associated nephropathy[Title/Abstract]) OR contrast media-induced nephropathy[Title/Abstract]) OR radiocontrast-induced nephropathy[Title/Abstract]) OR contrast-induced acute kidney injury[Title/Abstract]) OR contrast nephropathy[Title/Abstract]))) AND (((((((low-osmolar[Title/Abstract]) OR ((isoosmola*[Title/Abstract]) OR iso-osmola*[Title/Abstract])) OR contrast agent*[Title/Abstract]) OR ((((radiographic contrast[Title/Abstract]) OR radiocontrast media[Title/Abstract]) OR radiocontrast medium[Title/Abstract]) OR radiocontrast[Title/Abstract])) OR (((contrast media[Title/Abstract]) OR contrast medium[Title/Abstract]) OR contrast dye[Title/Abstract])) OR ((iotrolan[Title/Abstract]) OR Isovist[Title/Abstract])) OR ((iodixanol[Title/Abstract]) OR Visipaque[Title/Abstract])))** | [515](https://www.ncbi.nlm.nih.gov/pubmed/?cmd=HistorySearch&querykey=4) |

**Search criterion of Cochrane Central Register of Controlled Trials, from inception to May 31, 2017**

| **NO.** | **Query Results** | **Results** |
| --- | --- | --- |
| #1 | Contrast-induced nephropathy | 527 |
| #2 | Contrast induced nephropathy | 671 |
| #3 | contrast associated nephropathy | 291 |
| #4 | contrast media-induced nephropathy | 23 |
| #5 | radiocontrast-induced nephropathy | 33 |
| #6 | contrast-induced acute kidney injury | 219 |
| #7 | contrast nephropathy | 834 |
| #8 | #1 or #2 or #3 or #4 or #5 or #6 or #7 | 921 |
| #9 | renal failure | 14207 |
| #10 | renal dysfunction | 3849 |
| #11 | nephropathy* | 4949 |
| #12 | nephrotoxic* | 2451 |
| #13 | nephrotoxicity | 2218 |
| #14 | #9 or #10 or #11 or #12 or #13 | 21625 |
| #15 | #8 and #14 | 873 |
| #16 | random* | 721753 |
| #17 | Randomized Controlled Trials | 655703 |
| #18 | #16 or #17 | 721753 |
| #19 | #15 and #18 | 742 |
