# Supplementary material for: Contrast-induced nephropathy in patients with diabetes mellitus between iso- and low-osmolar contrast media: A meta-analysis of full-text prospective, randomized controlled trials
Source: PLoS One. 2018 Mar 20;13(3):e0194330. doi: 10.1371/journal.pone.0194330 (PMC5860737; doi:10.1371/journal.pone.0194330)
Supplement: S3 Table — (DOCX) [file pone.0194330.s003.docx]

**S3 Table. Quality assessment of Trials**

| **Studies** | **Type of Blinding** | **Method of Blinding Described and Appropriate** | **Randomization Process Described and Adequate** | **Adequate Concealed Allocation** | **Description of Withdrawals and Dropouts** | **Intention-to-Treatment at Analysis Performes** | **Important Baseline Differences Present** | **Inclusion and Exclusion Criteria Specified** | **Jadad Quality Score** |
| --- | --- | --- | --- | --- | --- | --- | --- | --- | --- |
| Aspelin 2003 | Double | Yes | Yes | Yes | Yes | NO | NO | Yes | 6 |
| Feldkamp 2006 | Double | Yes | Yes | NR | Yes | NR | NO | Yes | 6 |
| Jo 2006 | Double | Yes | Yes | NR | Yes | NR | NO | Yes | 5 |
| Solomon 2007 | Double | Yes | Yes | NR | Yes | NR | NO | Yes | 5 |
| Hardiek 2008 | Double | Yes | NR | NR | Yes | NR | NO | Yes | 4 |
| Kuhn 2008 | Double | NR | NR | NR | NR | NR | NO | Yes | 4 |
| Rudnick 2008 | Double | Yes | NR | NR | Yes | NR | NO | Yes | 4 |
| Chuang 2009 | Double | NR | NR | NR | Yes | NO | NO | Yes | 4 |
| Hernández 2009 | open-label | NO | NR | NR | Yes | NO | NO | Yes | 3 |
| Laskey 2009 | Double | Yes | NR | NR | Yes | Yes | NO | Yes | 5 |
| Shin 2011 | Double | Yes | Yes | NR | Yes | NO | NO | Yes | 5 |
| Chen 2012 | Double | YES | NR | NR | Yes | NR | NO | Yes | 5 |

NR = not reporte
